# Clinical and genetic characterization of Weaver syndrome: A case report of an *EZH2* mutation and review of the literature

**DOI:** 10.1097/MD.0000000000044080

**Published:** 2025-09-05

**Authors:** Luyu Ren, Liqing Jiang, Xiufang Jiang, Huabin Wang, Yan Li, Xueyun Ren

**Affiliations:** a Clinical Medical College of Jining Medical University (Affiliated Hospital), Jining, Shandong Province, China; b Department of Clinical Laboratory, Affiliated Hospital of Jining Medical University, Jining, Shandong Province, China; c Department of Pediatrics, Affiliated Hospital of Jining Medical University, Jining, Shandong Province, China; d Key Laboratory of Prevention and Control of Severe Infection in Children in Jining, Jining, Shandong Province, China.

**Keywords:** clinical diagnosis, *EZH2*, genetic testing, Weaver syndrome

## Abstract

**Rationale:**

Weaver syndrome is a rare congenital overgrowth disorder characterized by a wide spectrum of clinical manifestations that often overlap with other overgrowth syndromes. It is primarily caused by pathogenic variants in the *Enhancer of Zeste Homolog 2* (*EZH2*) gene on chromosome 7q36.1. Globally, fewer than 70 cases have been reported, with only a few documented in the Chinese population.

**Patient concerns:**

We report a 13-day-old Chinese male infant, born with macrosomia (birth weight: 5.04 kg), who was admitted for persistent neonatal jaundice. Physical examination and subsequent follow-up revealed accelerated postnatal growth and characteristic craniofacial features, including a broad forehead, hypertelorism, epicanthal folds, a flat nasal bridge, and low-set ears. His length, weight, and head circumference consistently plotted above the 97th percentile for his age. Additional findings included large hands and feet.

**Diagnoses:**

The child was ultimately diagnosed with “Weaver syndrome.”

**Interventions:**

DNA nanoballs were prepared with a universal sequencing reaction kit and subjected to paired-end sequencing on the MGISEQ-2000 platform. The resulting reads were aligned to the human reference genome hg19 (GRCh37). After removing PCR duplicates, single nucleotide polymorphisms and insertions/deletions were identified and annotated against established variant databases. The potential pathogenicity of the identified variants and their structural impact on the protein were evaluated using computational prediction tools. This analysis revealed a missense variant in the *EZH2* gene (NM_004456.4:c.2050C>T) in the proband, resulting in an arginine-to-cysteine substitution at codon 684 (p.Arg684Cys). In accordance with American College of Medical Genetics and Genomics guidelines, this variant was classified as pathogenic. Subsequent Sanger sequencing confirmed it as a de novo mutation.

**Outcomes:**

The patient received multidisciplinary guidance for neurodevelopmental, speech, and behavioral therapy. He remains under regular follow-up to monitor his growth and development.

**Lessons:**

This report documents a new case of Weaver syndrome in China harboring a de novo *EZH2* mutation, expanding the genotypic and phenotypic spectrum of this disorder in the Chinese population. Our findings underscore the critical role of genetic testing in achieving a definitive diagnosis for rare overgrowth syndromes, facilitating early intervention and appropriate management.

## 1. Introduction

Weaver syndrome (OMIM: 277590) is a rare congenital overgrowth syndrome defined by a triad of accelerated skeletal maturation, characteristic craniofacial features, and variable intellectual disability, which poses significant clinical and psychosocial challenges for patients and their families.^[1]^ Advances in molecular genetics have established that Weaver syndrome is predominantly caused by pathogenic variants in the *Enhancer of Zeste Homolog 2* (*EZH2*) gene (OMIM: 601573).^[2]^ While its exact incidence is unknown, the syndrome’s rarity is underscored by fewer than 70 genetically confirmed cases described globally and only 2 such cases previously reported in China.^[3–5]^ In this report, we present the clinical and genetic findings of the third case of Weaver syndrome in the Chinese population: a male infant who initially presented with macrosomia and recurrent neonatal jaundice and was subsequently diagnosed at 2.5 years of age through whole-exome sequencing. By integrating this case with a comprehensive literature review, we aim to expand the current understanding of the phenotypic spectrum, underlying genetic mechanisms, and management strategies for Weaver syndrome, thereby contributing to a more robust framework for its clinical practice.

## 2. Clinical data

The patient was a 13-day-old male infant transferred to our neonatal unit for management of persistent jaundice. He was born at 38 + 4 weeks of gestation via cesarean section, indicated for suspected macrosomia. His birth weight was 5.04 kg (>97th percentile). Apgar scores were 9 at both 1 and 5 minutes. Examination of the amniotic fluid, umbilical cord, and placenta revealed no abnormalities. Neonatal jaundice was first noted on day 3 of life and progressively worsened. Prior to transfer, investigations at a local hospital showed a total serum bilirubin of 338 μmol/L (indirect: 310 μmol/L). The infant was exclusively breastfed but demonstrated a weak suck. He had no history of fever or seizures. Stools were loose and yellow (5–6 per day), and urine output was normal.

Maternal and family history: The patient was the second live birth to a 32-year-old mother with 2 previous abortions and 1 unexplained fetal demise. Her obstetric history was significant for 2 prior spontaneous abortions and 1 unexplained fetal demise. The pregnancy was otherwise unremarkable, though a fetal ultrasound at 5 weeks of gestation reportedly indicated a fetus large for gestational age. The father was 37 years old. The parents were non-consanguineous and reported no family history of hereditary diseases or overgrowth syndromes.

Physical examination: The infant’s vital signs were as follows: temperature, 36.5°C; pulse, 140 beats/min; and respiratory rate, 42 breaths/min. Anthropometric measurements were: weight, 4.95 kg; length, 60 cm; and head circumference, 37 cm. The infant was alert and reactive with a hoarse cry, and his developmental and nutritional status appeared normal. Dysmorphic facial features were noted, including a high forehead, hypertelorism (wide-set eyes), a pointed chin, retrognathia, and low-set ears. The skin and mucous membranes were icteric, dry, and coarse, with mild desquamation. The remainder of the physical examination was unremarkable.

Diagnosis and treatment course: Upon admission, the infant was initiated on phototherapy and supportive care for hyperbilirubinemia. However, the jaundice recurred, necessitating the addition of phenobarbital to induce hepatic enzyme activity. The total duration of phototherapy was 32 hours. Subsequent thyroid function tests revealed the following: reverse T3 (rT3), 5.03 pmol/L; free T4 (FT4), 16.50 pmol/L; and thyroid-stimulating hormone, 23.7 mIU/L. Based on these findings, treatment with levothyroxine was initiated at a daily dose of 25 μg. An echocardiogram identified a 2-mm patent ductus arteriosus (PDA). The infant’s condition improved, and he was discharged after a 7-day hospitalization. The final discharge diagnoses were neonatal hyperbilirubinemia, neonatal hyperthyrotropinemia, PDA, and macrosomia.

**Figure 1.**
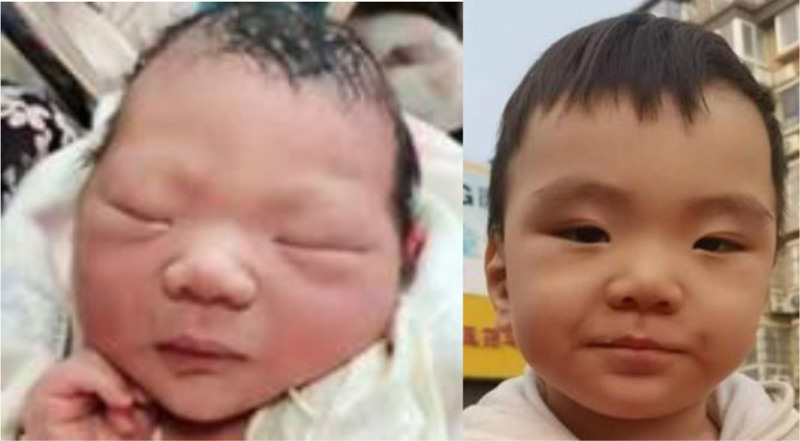
Over time, follow-up examinations noted the evolution of dysmorphic facial features, which included a broad forehead, hypertelorism, epicanthal folds, a flattened nasal bridge, and low-set ears.

**Figure 2.**
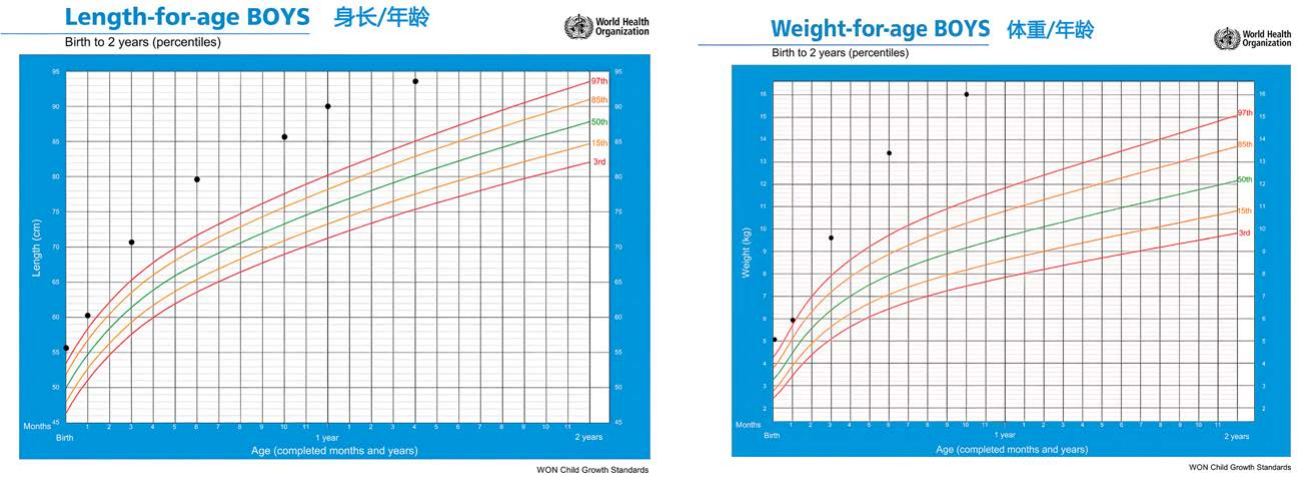
He presented with enlarged hands and feet, and his growth parameters – length, weight, and head circumference – persistently plotted above the 97th percentile on WHO growth charts. WHO = World Health Organization”.

**Figure 3.**
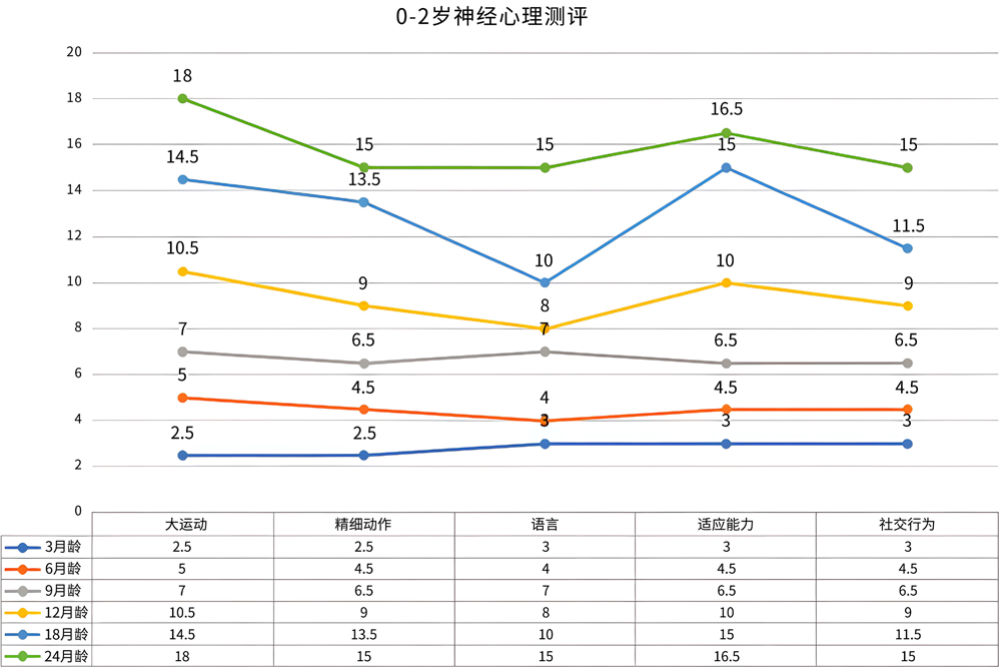
Neuropsychological assessment results of the Weaver syndrome patient from 0 to 2 yr of age, indicating delayed intellectual development.

Post-discharge follow-up and treatment: Post-discharge, the infant was monitored through regular follow-up appointments. He continued daily levothyroxine therapy, with the dosage titrated according to serial thyroid function tests. Over time, follow-up examinations noted the evolution of dysmorphic facial features, which included a broad forehead, hypertelorism, epicanthal folds, a flattened nasal bridge, and low-set ears (Fig. 1). The infant exhibited accelerated physical growth, consistently surpassing age-matched peers. He presented with enlarged hands and feet, and his growth parameters – length, weight, and head circumference – persistently plotted above the 97th percentile on WHO growth charts (Fig. 2). A notable developmental dissociation was observed: while motor development was within the normal range, significant delays were present in cognitive and language domains. He achieved motor milestones appropriately: rolling over at 6 months, sitting unsupported at 10 months (albeit with poor lateral balance), and walking independently at 12 months. In contrast, language development was delayed; at 2 years of age, his vocabulary was limited to single words, and by 2 years and 4 months, he could form short sentences, albeit with poor articulation. Serial neuropsychological assessments performed at 3, 6, 9, 12, 18, and 24 months consistently demonstrated these developmental delays (Fig. 3). Under the guidance of a pediatric rehabilitation specialist, the parents were instructed to implement a home-based training program targeting cognitive, language, and behavioral development.

**Figure 4.**
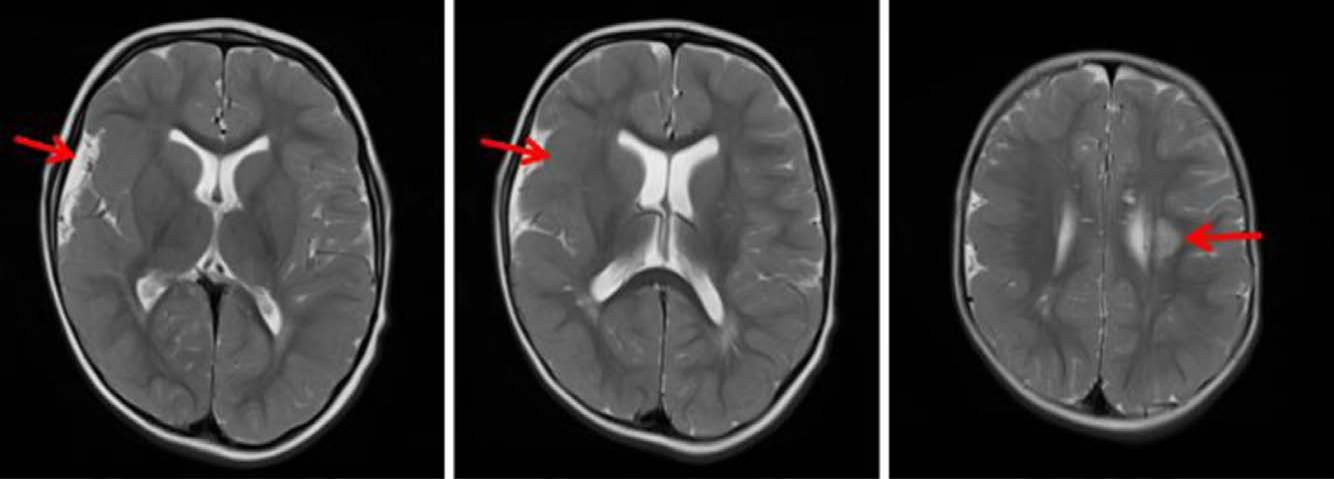
Focal gyral enlargement and broadening in the right cerebral hemisphere, gyral thickening in the left cerebral hemisphere, and hypomyelination in the left centrum semiovale (indicated by arrows).

At 2 years of age, cranial magnetic resonance imaging (MRI) revealed several abnormalities, including focal gyral enlargement and broadening in the right cerebral hemisphere, gyral thickening in the left cerebral hemisphere, and hypomyelination in the left centrum semiovale (Fig. 4).

To investigate the potential genetic etiology, we performed next-generation sequencing. After obtaining written informed consent from the patient’s legal guardians, peripheral blood samples (2 mL) were collected from the proband and his parents. Genomic DNA was subjected to targeted capture of exonic and adjacent splice-site regions. Library preparation was performed using combinatorial probe-anchor ligation (cPAS)-based chemistry to generate DNA nanoballs, followed by paired-end sequencing on an MGISEQ-2000 platform (BGI Tech Solutions Co., Ltd., Wuhan City, China).

**Figure 5.**
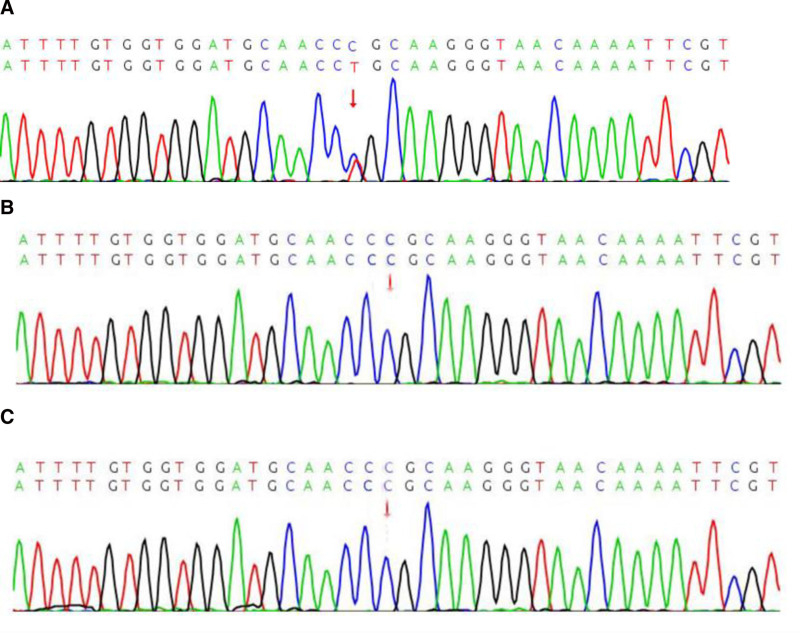
(A) This analysis identified a heterozygous missense variant in the *EZH2* gene: NM_004456.4:c.2050C>T (p.Arg684Cys) in the proband (indicated by the arrow). (B) Sanger sequencing result of the *EZH2* gene in the patient’s mother, showing the wild type sequence. (C) Sanger sequencing result of the *EZH2* gene in the patient’s father, showing the wild-type sequence. *EZH2 = Enhancer of Zeste Homolog 2*.

The sequencing data were processed through a standard bioinformatics pipeline. Briefly, reads were aligned to the human reference genome (hg19/GRCh37), and PCR duplicates were removed. Variant calling was performed to identify single nucleotide variants, small insertions/deletions, and copy number variations. The pathogenicity of identified variants was assessed in silico using multiple prediction tools, including REVEL, PolyPhen-2, and MutationTaster. The potential structural impact of the variant was further evaluated using PyMOL and AlphaFold protein modeling software. This analysis identified a heterozygous missense variant in the *EZH2* gene: NM_004456.4:c.2050C>T (p.Arg684Cys) in the proband (Fig. 5A). In accordance with the American College of Medical Genetics and Genomics and the Association for Molecular Pathology guidelines, this variant was classified as Pathogenic (evidence codes: PS4_Moderate, PM2, PM6_Strong, PP2, PP3). To confirm this finding and determine its origin, Sanger sequencing was performed, which validated the variant in the proband and demonstrated its absence in both parents, establishing it as a de novo event (Fig. 5B, C). Based on the combination of the characteristic clinical phenotype and the identification of a pathogenic de novo EZH2 variant, the patient was definitively diagnosed with Weaver syndrome. He remains under routine clinical follow-up. This study was approved by the Medical Research Ethics Committee of the Affiliated Hospital of Jining Medical University.

## 3. Discussion

Weaver syndrome was first described in 1974 by Weaver et al, who reported 2 unrelated male infants with a constellation of similar clinical features: accelerated somatic growth, advanced bone age, distinctive craniofacial dysmorphism, a hoarse and low-pitched cry, hypertonia, and camptodactyly^[1]^ (Table 1). These features have since formed the basis for its clinical recognition. The condition was formally established as a distinct clinical entity by Ardinger et al.^[6]^ Despite these foundational reports, the underlying etiology, pathogenic mechanisms, and the full spectrum of genotypic and phenotypic features of Weaver syndrome are not yet completely understood (Table 2). This report details the clinical course, genetic findings, and diagnostic process of a patient with Weaver syndrome, aiming to contribute to a more comprehensive understanding of this rare disorder.

**Table 1:** Clinical manifestations of Weaver syndrome.

Year of publication	Paper title	Growth and development	Craniofacial features	Limbs and skeleton	Neurological and developmental aspects	Other manifestations	Molecular genetic features
1974	A new overgrowth syndrome with accelerated skeletal maturation, unusual facies, and camptodactyly	Prenatal/postnatal overgrowth with accelerated, disharmonic bone age (carpal bones prominently advanced)	Distinctive craniofacial features including hypertelorism, large ears, and relative micrognathia	Common limb/skeletal anomalies with individual-specific deformities and limited joint extension	Hypertonia and hoarse cry with motor delay in 1 patient, normal development in the other	Loose skin, umbilical hernia, and patient-specific anomalies with mild amino acid elevation	–
1980	The Weaver-Smith syndrome	Overgrowth trend with accelerated, dysharmonic skeletal maturation and abnormal metaphyses/epiphyses, plus neonatal transient hyperbilirubinemia	Distinctive craniofacial/sensory features including Pierre Robin anomalad, large low-set ears, and convergent strabismus	Limb anomalies (large hands/feet, camptodactyly, etc) with bilateral cryptorchidism and ureteral dystonia	–	–	–
1981	Accelerated bone maturation syndrome of the Weaver type	–	Craniofacial anomalies including dolichomacrocephaly, hypertelorism, retrognathia, and large dysplastic low-set ears	Skin (excessive loose skin) and limb abnormalities with camptodactyly, muscular hypertonia, and limited joint movement	–	Thoracic deformity, heart murmur, and multiple genital/inguinal anomalies	–
1986	Further delineation of Weaver syndrome	Overgrowth (birth or postnatal) with dysharmonic skeletal maturation and widened distal long bones	Distinctive craniofacial features including hypertelorism, large ears, and relative micrognathia	Limb anomalies, limited joint mobility, loose skin, and common hernias	Developmental delay, abnormal muscle tone, hoarse voice, and possible adult spasticity	Early tooth eruption, various anomalies, and specific radiographic findings	–
1985	Weaver’s syndrome – primordial excessive growth velocity: a case report	Marked accelerated growth with dysharmonic skeletal maturation and abnormal long bone metaphyses/epiphyses	Distinctive craniofacial features including hypertelorism, micrognathia, and large dysplastic ears	Limb deformities, limited joint extension, loose skin, and lumbar kyphosis	–	Hoarse voice, hernias, CNS hypertonia, and recurrent respiratory infections with other systemic manifestations	–
1981	The Weaver syndrome: A rare type of primordial overgrowth	Accelerated postnatal growth (head circumference prominent) with dysharmonic skeletal maturation in both males	Shared craniofacial features with individual variations in both cases	Foot deformities and nail abnormalities in both, with webbed neck only in Case 1	-	Shared abnormal voices, hernias, developmental delay with individual neurological/cardiac differences	–
1996	Novel findings in a patient with Weaver or a Weaver-like syndrome	Postnatal accelerated growth with advanced skeletal maturation and specific bone abnormalities	Distinctive craniofacial features including hypertelorism and strabismus with hoarse cry	Limb/skin anomalies with toe syndactyly and bilateral cryptorchidism	Severe neurodevelopmental retardation with hypotonia and postural difficulties	Brain MRI anomalies with normal karyotype, biochemical, and metabolic findings	–
1989	A new autosomal recessive disorder resembling Weaver syndrome	Prenatal overgrowth with contrasting postnatal growth (acceleration vs retardation) and skeletal maturation patterns	Shared craniofacial features with distinct individual variations in both cases	Common limb/skin anomalies with case-specific digital and skin variations	Shared neurological issues with varying degrees of psychomotor retardation	Shared dental, genital, and brain imaging abnormalities	–
1991	Weaver syndrome in 2 Japanese children	Postnatal growth acceleration with dysharmonic skeletal maturation and femoral metaphyseal splaying	Shared craniofacial features with individual variations (ptosis in Case 1, short neck in Case 2)	Shared narrow shoulders, abnormal chest, and hypertonia with variable limb findings	Mild neurodevelopmental delay with social withdrawal and friendly demeanor	Elevated total cholesterol with normal chromosomes and other laboratory findings	–
1992	Weaver syndrome	Prenatal/postnatal overgrowth with dysharmonic skeletal maturation and flared long bone metaphyses	Consistent craniofacial features with age-related facial shape changes, Sotos-like phenotype	Limb anomalies, joint limitation, and common genital/umbilical hernias	Developmental delays, variable intellectual function, behavioral issues, and hoarse voice	Normal karyotypes, mostly normal endocrine function, and specific imaging findings	–
1987	A girl with the Weaver syndrome	Postnatal growth acceleration with advanced skeletal maturation and widened distal femora	Distinctive craniofacial features with dental anomalies and hoarse voice	Limb/trunk anomalies with hypertonia, joint limitation, and spinal deformities	Severe developmental delay with brain structural abnormalities on CT	Recurrent respiratory issues, laryngomalacia, and transient umbilical hernia	–
1990	A Japanese male infant with the Weaver syndrome	Prenatal/postnatal overgrowth with dysharmonic skeletal maturation and splayed long bone metaphyses	Distinctive craniofacial features with hoarse low-pitched cr	Limb anomalies with loose neck skin and no joint extension limitation	Mild developmental delay and hypertonia	Normal karyotype with thyroid function abnormalities and otherwise normal findings	–
1989	Weaver syndrome: The changing phenotype in an adult	Accelerated postnatal growth with advanced skeletal maturation persisting into adulthood	Childhood-specific craniofacial features (Weaver-like) with adult attenuation	Persistent camptodactyly with childhood-specific palate/hernia/clonus	Mild intellectual impairment with transient hypotonia	Myopia with normal genetics and no familial syndrome manifestations	–
1988	Retarded skeletal maturation in weaver syndrome	Sustained overgrowth with retarded skeletal maturation and specific bone anomalies	Distinctive craniofacial features with maternal phenotypic overlap	Limb anomalies, skin redundancy, and multiple hernias with cryptorchidism	Neurodevelopmental delay with hypotonia and cerebral atrophy on imaging	Normal labs and karyotype with diaphragmatic hernia	–
2010	Acute lymphoblastic leukemia in Weaver syndrome	Sustained overgrowth with accelerated, dysharmonic skeletal maturation	Distinctive craniofacial features with a hoarse voice	Limb/trunk anomalies including large extremities, joint hyperlaxity, and vertebral beaking	Developmental delay (speech/motor) with brain MRI abnormalities	Cafe au lait spot, ALL with 47,XXX karyotype, and normal genetic screening	–
1999	A probable case of familial Weaver syndrome associated with neoplasia	Persistent overgrowth with accelerated, disharmonious skeletal maturation	Distinctive craniofacial features including round face and “Cupid’s bow” lips	Limb anomalies with hypertonia and abnormal gait	Developmental delays (notably speech) with later motor/social deficits and obsessive traits	Normal genetic and metabolic screening results	–
1993	Twins and their mildly affected mother with Weaver syndrome	Postnatal growth acceleration with advanced skeletal maturation and distal femoral abnormalities	Distinctive craniofacial features with a hoarse low-pitched voice	Limb/skin anomalies with shared hyperhidrosis and boy-specific features	Neurodevelopmental delay, low IQ, and persistent hypertonia	Boy with surgical corrections; normal laboratory and genetic tests	–
2000	Cervical spine anomalies and tumors in Weaver syndrome	Marked postnatal overgrowth with significantly accelerated skeletal maturation in both cases	Common craniofacial/trunk features with individual variations in facial and joint findings	Shared finger joint contractures and limited mobility with case-specific joint anomalies	Developmental delays with variable cognitive impairment severity	Shared cervical spine and airway issues with individual-specific extra manifestations	–
1980	The syndromes of Marshall and Weaver	Prenatal/postnatal overgrowth with dysharmonic skeletal maturation and distal long bone widening	Distinctive craniofacial features with a hoarse low-pitched voice	Limb anomalies, joint extension limitation, and excessive loose skin	Hypertonia, excessive appetite, and psychomotor delay with possible mental retardation	Hernias, inverted nipples, sparse hair, and variable joint/skeletal abnormalities	–
2009	Weaver syndrome: A report of a rare genetic syndrome	Prenatal-onset overgrowth with accelerated skeletal maturation and distal long bone abnormalities	Distinctive craniofacial features including hypertelorism and a low-pitched voice	Limb anomalies, joint extension limitation, and distal long bone/foot deformities	Presence of psychomotor retardation	Loose skin, positive family history, and normal blood tests/karyotype	–
1997	Autosomal dominant inheritance of Weaver syndrome	Markedly accelerated postnatal growth with advanced bone age and increased leg length	Classic Weaver syndrome craniofacial features, paternal phenotypic overlap	Isolated nail anomalies without characteristic Weaver syndrome digital findings	Hypotonia, developmental delays (motor/language), and clumsiness requiring special care	–	–
2008	Treatment of macroglossia in a child with Weaver syndrome	Prenatal/postnatal overgrowth with dysharmonic skeletal maturation (carpophalangeal advancement) at 3 yr	Distinctive craniofacial features dominated by macroglossia and relative micrognathia	Limb/skin anomalies with toe/nail defects and corrected cryptorchidism	Motor/language delays with suspected mental handicap	Congenital hypothyroidism, dental anomalies, normal karyotype, and successful glossectomy outcomes	–
1998	Weaver syndrome: Autosomal dominant inheritance of the disorder	Persistent overgrowth from birth with accelerated, dysharmonic skeletal maturation in both siblings	Common craniofacial features with individual-specific anomalies in both siblings	Shared limb/skin anomalies with gender-specific genital and digital variations	Neurodevelopmental delays with more severe neurological findings in the sister	Siblings with diverse systemic anomalies and paternal phenotypic overlap	–
2003	Spectrum of NSD1 mutations in Sotos and Weaver syndromes	Overgrowth, macrocephaly, and accelerated skeletal maturation with distal long bone widening in all patients	Characteristic craniofacial features with deep-set nails in mutation-positive cases	Consistent limb anomalies with variable cutis laxa and metaphyseal widening	Universal moderate speech delay with rare feeding difficulties	–	De novo NSD1 mutations (diverse types) in half of the patients
2008	Weaver syndrome and neuroblastoma	Accelerated postnatal growth with significantly advanced skeletal maturation	Distinctive craniofacial features with hemangioma and hoarse voice	Isolated limb/oral anomalies of prominent fingertip pads and narrow palate	Bilateral cortical dysplasia (right-sided predominance) on brain MRI	Multiple congenital anomalies with stage IV neuroblastoma and normal genetic testing	–
2004	Hormonal and genetical assessment of a Japanese girl with Weaver syndrome	Sustained accelerated growth with dysharmonic skeletal maturation and active bone metabolism	Distinctive craniofacial features including macrocephaly and a hoarse low-pitched voice	Limb contractures, hypertonia, and repaired submucosal cleft palate	Mild language delay with infancy airway issues and specific behavioral traits	Normal genetics/endocrinology with advanced bone mineral density	–
1998	Familial neurofibromatosis type 1 associated with an overgrowth syndrome resembling Weaver syndrome	Post-6-mo overgrowth with accelerated skeletal maturation, and maternal tall stature	Shared coarse facial features with age-related changes in the boy and maternal NF1 stigmata	Limb/skin anomalies in the boy and maternal hernia with adult neurofibromas	Neurodevelopmental delays and optic glioma in the boy, mild learning difficulties in the mother	Confirmed NF1 in both via 17q11.2 deletion, with normal endocrine/karyotype in the boy	–
2002	The difficulty of height prediction in Weaver syndrome	Persistent accelerated growth with dysharmonic skeletal maturation and delayed puberty	Distinctive craniofacial features including macrocephaly and a hoarse low-pitched voice	Multiple limb anomalies, joint limitations, and loose skin with narrow shoulders	Severe psychomotor retardation with neonatal hypotonia and feeding issues	Structural anomalies, growth hormone axis dysfunction, and hormone-induced puberty	–
1996	Propositus with Weaver syndrome and his mildly-affected mother: Implication of nontraditional inheritance?	Sustained overgrowth with accelerated, dysharmonic skeletal maturation (carpal advancement)	Distinctive craniofacial features with maternal phenotypic overlap in size/auricles	Shared skeletal anomalies (coxa valga, tibial changes) in both child and mother	Normal psychomotor development with age-appropriate milestones	Transient neonatal jaundice, normal labs, and no classic Weaver syndrome traits	–
2016	Anesthetic management of a patient with Weaver syndrome undergoing emergency evacuation of extradural hematoma: A case report and review of the literature	Marked accelerated growth with early diagnosis of hypotonia and developmental delay	Intubation-relevant craniofacial anomalies and history of umbilical hernia repair	–	Developmental delay, hypotonia, and post-fall intracranial hemorrhage with herniation	–	–
1990	Weaver syndrome and instability of the upper cervical spine	Birth overgrowth with accelerated, dysharmonic skeletal maturation	Distinctive craniofacial anomalies with a hoarse low-pitched voice	Limb anomalies and cervical spine instability with spinal cord compromise	Severe neurodevelopmental delay with hypertonia, improving postoperatively	Cardiac anomaly, past neuroblastoma, and cervical spine MRI-confirmed cord compression	–
2003	NSD1 mutations are the major cause of Sotos syndrome and occur in some cases of Weaver syndrome but are rare in other overgrowth phenotypes	Consistent prenatal/postnatal overgrowth with dysharmonic skeletal maturation	Uniform distinctive craniofacial features including a hoarse voice	Consistent limb/skin anomalies with metaphyseal flaring and joint limitation	Universal developmental delay with variable intellectual disability	De novo NSD1 mutations in specific exons in ~40% of patients	–
2013	Weaver syndrome and EZH2 mutations: Clarifying the clinical phenotype	Consistent overgrowth with advanced, sometimes disharmonious bone age and increased head circumference	Childhood-specific craniofacial features, attenuating with age, overlapping with Sotos syndrome	Common limb/skin anomalies with variable skeletal deformities and adult-specific finger posture	Prevalent intellectual disability, coordination issues, and variable tone abnormalities	Variable nonspecific manifestations including hernias, voice changes, and rare tumors	Predominantly de novo EZH2 mutations, with missense clustering in the SET domain
2016	EZH2 mutation in an adolescent with Weaver syndrome developing acute myeloid leukemia and secondary hemophagocytic lymphohistiocytosis	Prenatal/postnatal overgrowth with significantly elevated height in adolescence	Distinctive craniofacial features with cleft palate and surgically managed thoracic hyperkyphosis	–	Perceptual/speech deficits with preserved cognitive ability	Fatal AML with FLT3-ITD and secondary HLH without infectious triggers	Germline EZH2 p.Pro132Leu mutation in the catalytic D1 domain
2016	Novel EED mutation in patient with Weaver syndrome	Sustained overgrowth from large-for-gestational-age birth with significantly advanced bone age	Distinctive craniofacial features including ear anomalies and post-ptosis surgery changes	Multiple limb/skin anomalies with joint issues and skeletal abnormalities	Global developmental delay with behavioural issues and speech/gait abnormalities	Multiple congenital/systemic anomalies with extensive surgical history	Novel pathogenic de novo EED p.Arg302Gly variant
2018	EZH2 mutations found in the Weaver overgrowth syndrome cause a partial loss of H3K27 histone methyltransferase activity	Accelerated childhood growth with advanced bone age despite normal parental stature	Weaver syndrome-like craniofacial features including macrocephaly and large ears	Isolated limb anomaly of large hands with thin, deep-set nails	Moderate global neurodevelopmental delays	Normal endocrine evaluations with negative Sotos syndrome and genomic array findings	De novo EZH2 p.Val626Met mutation with partial histone methyltransferase activity loss
2011	Germline mutations in the oncogene EZH2 cause Weaver syndrome and increased human height	Consistent prenatal/postnatal overgrowth with marked height excess and relatively milder head circumference increase	Subtle but recognizable typical craniofacial features prone to underdiagnosis	–	Predominantly mild to moderate learning disabilities without severe intellectual disability	Occasional malignancies and autosomal dominant inheritance in some families	EZH2 mutations (mostly functional domain missense) with mixed de novo and familial inheritance
2012	Mutations in EZH2 cause Weaver syndrome	Marked prenatal/postnatal overgrowth with advanced, dysharmonic skeletal maturation	Distinctive craniofacial features including macrocephaly and “stuck-on chin”	Consistent limb anomalies with variable skeletal/skin manifestations	Neurodevelopmental impairments with variable neurological findings	Diverse systemic anomalies including endocrine and cardiac issues	De novo EZH2 mutations in functional domains affecting histone methyltransferase activity
2017	Mutations in genes encoding polycomb repressive complex 2 subunits cause Weaver syndrome	Persistent prenatal/postnatal overgrowth with accelerated, dysharmonic skeletal maturation	Distinctive craniofacial features with a hoarse voice and occasional palatal malformations	Consistent limb anomalies with variable skin and skeletal abnormalities	Neurodevelopmental delays with variable intellectual disability and coordination/tone issues	Predominant hernias with occasional cardiac, oncologic, or brain imaging anomalies	Linked to de novo mutations in NSD1/EZH2 (some affecting SET domain)
2013	Weaver syndrome and defective cortical development: A rare association	Postnatal growth acceleration with markedly advanced bone age	Typical Weaver syndrome craniofacial features including hypertelorism and retrognathia	Asymmetric lower extremities with left-sided neurological signs and umbilical hernia	Left-sided weakness with right hemispheric polymicrogyria and normal developmental milestones	Isolated neonatal transient hyperbilirubinemia with conservative management	Recurrent de novo EZH2 p.Glu745Lys mutation not found in reference databases
2018	Novel de novo mutation affecting 2 adjacent aminoacids in the *EED* gene in a patient with Weaver syndrome	Accelerated postnatal growth with significantly advanced bone age	Fine hair, wide prominent forehead, large ears, hypertelorism, epicanthic folds, almond-shaped palpebral fissures, prominent deep philtrum, open mouth, widely spaced teeth, retrognathia, pointed chin (Weaver syndrome facies); hoarse voice	Limb/trunk anomalies including large extremities, club feet, and hypotonia	Delayed development, moderate intellectual disability, hearing loss, and mild ventricular enlargement	Neonatal cryptorchidism with a de novo EED mutation (normal EZH2)	Neonatal cryptorchidism with a de novo EED mutation (normal EZH2)
2019	EED and EZH2 constitutive variants: A study to expand the Cohen-Gibson syndrome phenotype and contrast it with Weaver syndrome	Sustained prenatal/postnatal overgrowth with advanced bone age across the lifespan	Characteristic facies (broad forehead, frontal bossing, telecanthus, almond-shaped palpebral fissures, large ears, retrognathia, pointed chin with horizontal crease) with partial age-related changes	Frequent limb contractures and spinal deformities with occasional large extremities	Predominantly mild to moderate intellectual disability with common tone abnormalities	Prevalent umbilical hernias with occasional tumors, cardiac anomalies, or cryptorchidism	–
2021	Anovel *EZH2* gene variant in a case of Weaver syndrome with postaxial polydactyly	Marked prenatal/postnatal overgrowth with non-accelerated bone age and eventual epiphyseal fusion	Macrocephaly, large bifrontal diameter, flattened occiput, deep-set eyes, large ears, down-slanting palpebral fissures, strabismus, hypertelorism (>97th centile), prominent chin	Multiple limb/skeletal anomalies including polydactyly, syndactyly, and spinal deformities	Mild intellectual disability with language delay and asocial traits	Cardiac and ophthalmological anomalies with normal endocrine and karyotype	Likely pathogenic de novo EZH2 p.His240Arg variant
1983	Siblings with Weaver syndrome	Siblings with prenatal overgrowth, with patient 2 showing post-3-yr growth deceleration and advanced bone age	Shared hypertelorism and micrognathia with sibling-specific craniofacial variations	Limb/skin anomalies with skeletal abnormalities in patient 2	Severe neurodevelopmental delay with structural brain abnormalities in patient 2	Shared respiratory/cry anomalies with Patient 1 having fatal cardiac/infectious complications	–
2000	Excessive growth in a girl with Weaver syndrome	Prenatal/postnatal overgrowth with advanced bone age	Characteristic facies including broad forehead, hypertelorism, large ears, long philtrum, and relative micrognathia	Typical limb/skeletal anomalies with potential joint mobility issues	Psychomotor retardation of varying severity	Additional features include a low-pitched voice and loose skin	–
2021	A novel *EZH2* gene variant in a case of Weaver syndrome with postaxial polydactyly	Marked prenatal/postnatal overgrowth with non-accelerated bone age and eventual epiphyseal fusion	Weaver syndrome-typical craniofacial features including macrocephaly and hypertelorism	Multiple limb/skeletal anomalies including polydactyly, syndactyly, and spinal deformities	Mild intellectual disability with language delay and asocial traits	Cardiac and ophthalmological anomalies with normal endocrine and karyotype	Likely pathogenic de novo EZH2 p.His240Arg variant
1983	The Weaver syndrome in a girl	Persistent postnatal overgrowth with progressively advanced bone age	Typical Weaver syndrome craniofacial features including telecanthus and micrognathia	Limb polydactyly, skeletal anomalies, and loose skin with thickened acral pads	Mild hypertonia and motor delay with preserved social abilities	Hoarse cry, low-pitched voice, and excessive appetite without camptodactyly	–
1985	Brief clinical report: Weaver syndrome with pes cavus	Postnatal overgrowth with markedly advanced bone age	Distinctive facies (slightly downslanting palpebral fissures, epicanthic folds, flat nasal root, telecanthus, flaring eyebrows, broad forehead, small chin); flat occiput, short neck; normal ears/teeth	Limb anomalies (pes cavus, nail hypoplasia) and skull wormian bones	Developmental delays with persistent gross motor/expressive language deficits and hyperactive behavior	Congenital facial anomalies, repaired hernia, and normal investigations	–
1991	Weaver syndrome: A case without early overgrowth and review of the literature	Post-feeding weight overgrowth with isolated carpal bone age acceleration despite normal length	Distinctive craniofacial anomalies including marked hypertelorism and micrognathia	Limb anomalies with disharmonic skeletal maturation (advanced carpal bones)	Severe neurodevelopmental impairment with intractable seizures and encephalopathy	Multiple systemic complications leading to early death with significant autopsy findings	Normal female karyotype with no chromosomal abnormalities
2001	Weaver syndrome with neuroblastoma and cardiovascular anomalies	Prenatal and postnatal overgrowth with distinctive skeletal anomalies	Distinctive craniofacial features including marked hypertelorism and large ears	Multiple limb and skeletal anomalies with loose neck skin	Mild speech delay with otherwise age-appropriate development	Multiple anomalies including regressed neuroblastoma, cardiac defects, and neonatal hypoglycemia	–
2010	Weaver syndrome associated with bilateral congenital hip and unilateral subtalar dislocation	Overgrowth tendency with accelerated skeletal maturation typical of Weaver syndrome	Typical craniofacial features including large skull and hypertelorism	Multiple limb and skeletal anomalies with significant foot deformities	Mild psychomotor delay with hypotonia and inability to walk	Pulmonary issues with familial autosomal dominant inheritance pattern	–
2015	Oral, radiographical, and clinical findings in Weaver syndrome: a case report	Marked prenatal/postnatal overgrowth with accelerated skeletal maturation, leading to wheelchair dependence	Typical craniofacial features including large head and hypertelorism	Limb anomalies with hypertonia and polydactyl trait	Neurodevelopmental impairments including autism, seizures, and intellectual disability with behavioral deterioration	Multiple oral/maxillofacial anomalies with severe periodontal issues and distinctive dental radiologic features	–
2023	Expanding the phenotypic and genotypic spectrum of Weaver syndrome: A missense variant of the *EZH2* gene	Marked prenatal/postnatal overgrowth in a premature infant, persisting postnatally	Typical craniofacial features including hypertelorism and micrognathia with thin hair	Limb/skin anomalies with mixed tone abnormalities and reflex hyperactivity	Neurological sequelae including mild intellectual disability and periventricular leukomalacia	Maternal/fetal complications with a likely pathogenic de novo EZH2 variant and gingival hypertrophy	Maternal/fetal complications with a likely pathogenic de novo EZH2 variant and gingival hypertrophy
2021	A case of Weaver syndrome caused by *EZH2* gene variation	Significant overgrowth with accelerated skeletal maturation and rapid postnatal growth	Typical craniofacial features including hypertelorism and a protruding forehead	Limb/skeletal features with finger pads, gait issues, and normal muscle function	Neurodevelopmental impairments with speech issues and poor intellectual response	Multiple surgical/medical histories with neurological and cardiac imaging abnormalities	Pathogenic de novo EZH2 p.K574E variant identified via whole-exome sequencing
2024	A case of Weaver syndrome caused by *EZH2* gene variation and literature review	Mild growth delay (possibly infection-related) consistent with potential Weaver syndrome growth traits	Typical craniofacial features including hypertelorism and large ears	Limb/skin anomalies with early fontanel closure and increased lower limb tone	Global developmental delay with feeding/swallowing issues and normal neuroimaging	Severe respiratory complications (with new diaphragmatic anomaly) and minor cardiac defect	De novo likely pathogenic EZH2 frameshift variant identified via whole-exome sequencing

AML = acute myeloid leukemia, CNS = central nervous system, CT = computed tomography, *EZH2 = Enhancer of Zeste Homolog 2*, HLH = hemophagocytic lymphohistiocytosis, ITD = internal tandem duplication, MRI = magnetic resonance imaging, NSD1 = nuclear receptor binding SET domain protein 1.

**Table 2:** Clinical manifestations of Weaver syndrome caused by EZH2.

Year of publication	Paper title	Growth and development	Craniofacial features	Limbs and skeleton	Neurological and developmental aspects	Other manifestations	Molecular genetic features
2003	Spectrum of NSD1 mutations in Sotos and Weaver syndromes	Overgrowth, macrocephaly, and accelerated skeletal maturation with distal long bone widening in all patients	Characteristic craniofacial features with deep-set nails in mutation-positive cases	Consistent limb anomalies with variable cutis laxa and metaphyseal widening	Universal moderate speech delay with rare feeding difficulties	–	De novo NSD1 mutations (diverse types) in half of the patients
2013	Weaver syndrome and EZH2 mutations: Clarifying the clinical phenotype	Consistent overgrowth with advanced, sometimes disharmonious bone age and increased head circumference	Childhood-specific craniofacial features, attenuating with age, overlapping with Sotos syndrome	Common limb/skin anomalies with variable skeletal deformities and adult-specific finger posture	Prevalent intellectual disability, coordination issues, and variable tone abnormalities	Variable nonspecific manifestations including hernias, voice changes, and rare tumors	Predominantly de novo EZH2 mutations, with missense clustering in the SET domain
2016	EZH2 mutation in an adolescent with Weaver syndrome developing acute myeloid leukemia and secondary hemophagocytic lymphohistiocytosis	Prenatal/postnatal overgrowth with significantly elevated height in adolescence	Distinctive craniofacial features with cleft palate and surgically managed thoracic hyperkyphosis	–	Perceptual/speech deficits with preserved cognitive ability	Fatal AML with FLT3-ITD and secondary HLH without infectious triggers	Germline EZH2 p.Pro132Leu mutation in the catalytic D1 domain
2016	Novel EED mutation in patient with Weaver syndrome	Sustained overgrowth from large-for-gestational-age birth with significantly advanced bone age	Distinctive craniofacial features including ear anomalies and post-ptosis surgery changes	Multiple limb/skin anomalies with joint issues and skeletal abnormalities	Global developmental delay with behavioral issues and speech/gait abnormalities	Multiple congenital/systemic anomalies with extensive surgical history	Novel pathogenic de novo EED p.Arg302Gly variant
2018	EZH2 mutations found in the Weaver overgrowth syndrome cause a partial loss of H3K27 histone methyltransferase activity	Accelerated childhood growth with advanced bone age despite normal parental stature	Weaver syndrome-like craniofacial features including macrocephaly and large ears	Isolated limb anomaly of large hands with thin, deep-set nails	Moderate global neurodevelopmental delays	Normal endocrine evaluations with negative Sotos syndrome and genomic array findings	De novo EZH2 p.Val626Met mutation with partial histone methyltransferase activity loss
2011	Germline mutations in the oncogene EZH2 cause Weaver syndrome and increased human height	Consistent prenatal/postnatal overgrowth with marked height excess and relatively milder head circumference increase	Subtle but recognizable typical craniofacial features prone to underdiagnosis	–	Predominantly mild to moderate learning disabilities without severe intellectual disability	Occasional malignancies and autosomal dominant inheritance in some families	EZH2 mutations (mostly functional domain missense) with mixed de novo and familial inheritance
2012	Mutations in EZH2 cause Weaver syndrome	Marked prenatal/postnatal overgrowth with advanced, dysharmonic skeletal maturation	Distinctive craniofacial features including macrocephaly and “stuck-on chin”	Consistent limb anomalies with variable skeletal/skin manifestations	Neurodevelopmental impairments with variable neurological findings	Diverse systemic anomalies including endocrine and cardiac issues	De novo EZH2 mutations in functional domains affecting histone methyltransferase activity
2017	Mutations in genes encoding polycomb repressive complex 2 subunits cause Weaver syndrome	Persistent prenatal/postnatal overgrowth with accelerated, dysharmonic skeletal maturation	Distinctive craniofacial features with a hoarse voice and occasional palatal malformations	Consistent limb anomalies with variable skin and skeletal abnormalities	Neurodevelopmental delays with variable intellectual disability and coordination/tone issues	Predominant hernias with occasional cardiac, oncologic, or brain imaging anomalies	Linked to de novo mutations in NSD1/EZH2 (some affecting SET domain)
2013	Weaver syndrome and defective cortical development: A rare association	Postnatal growth acceleration with markedly advanced bone age	Typical Weaver syndrome craniofacial features including hypertelorism and retrognathia	Asymmetric lower extremities with left-sided neurological signs and umbilical hernia	Left-sided weakness with right hemispheric polymicrogyria and normal developmental milestones	Isolated neonatal transient hyperbilirubinemia with conservative management	Recurrent de novo EZH2 p.Glu745Lys mutation not found in reference databases
2018	Novel de novo mutation affecting 2 adjacent aminoacids in the *EED* gene in a patient with Weaver syndrome	Accelerated postnatal growth with significantly advanced bone age	Fine hair, wide prominent forehead, large ears, hypertelorism, epicanthic folds, almond-shaped palpebral fissures, prominent deep philtrum, open mouth, widely spaced teeth, retrognathia, pointed chin (Weaver syndrome facies); hoarse voice	Limb/trunk anomalies including large extremities, club feet, and hypotonia	Delayed development, moderate intellectual disability, hearing loss, and mild ventricular enlargement	Neonatal cryptorchidism with a de novo EED mutation (normal EZH2)	Neonatal cryptorchidism with a de novo EED mutation (normal EZH2)
2019	EED and EZH2 constitutive variants: A study to expand the Cohen-Gibson syndrome phenotype and contrast it with Weaver syndrome	Sustained prenatal/postnatal overgrowth with advanced bone age across the lifespan	Characteristic facies (broad forehead, frontal bossing, telecanthus, almond-shaped palpebral fissures, large ears, retrognathia, pointed chin with horizontal crease) with partial age-related changes	Frequent limb contractures and spinal deformities with occasional large extremities	Predominantly mild to moderate intellectual disability with common tone abnormalities	Prevalent umbilical hernias with occasional tumors, cardiac anomalies, or cryptorchidism	–
2021	Anovel *EZH2* gene variant in a case of Weaver syndrome with postaxial polydactyly	Marked prenatal/postnatal overgrowth with non-accelerated bone age and eventual epiphyseal fusion	Macrocephaly, large bifrontal diameter, flattened occiput, deep-set eyes, large ears, down-slanting palpebral fissures, strabismus, hypertelorism (>97th centile), prominent chin	Multiple limb/skeletal anomalies including polydactyly, syndactyly, and spinal deformities	Mild intellectual disability with language delay and asocial traits	Cardiac and ophthalmological anomalies with normal endocrine and karyotype	Likely pathogenic de novo EZH2 p.His240Arg variant
2021	A novel *EZH2* gene variant in a case of Weaver syndrome with postaxial polydactyly	Marked prenatal/postnatal overgrowth with non-accelerated bone age and eventual epiphyseal fusion	Weaver syndrome-typical craniofacial features including macrocephaly and hypertelorism	Multiple limb/skeletal anomalies including polydactyly, syndactyly, and spinal deformities	Mild intellectual disability with language delay and asocial traits	Cardiac and ophthalmological anomalies with normal endocrine and karyotype	Likely pathogenic de novo EZH2 p.His240Arg variant
1991	Weaver syndrome: A case without early overgrowth and review of the literature	Post-feeding weight overgrowth with isolated carpal bone age acceleration despite normal length	Distinctive craniofacial anomalies including marked hypertelorism and micrognathia	Limb anomalies with disharmonic skeletal maturation (advanced carpal bones)	Severe neurodevelopmental impairment with intractable seizures and encephalopathy	Multiple systemic complications leading to early death with significant autopsy findings	Normal female karyotype with no chromosomal abnormalities
2023	Expanding the phenotypic and genotypic spectrum of Weaver syndrome: A missense variant of the *EZH2* gene	Marked prenatal/postnatal overgrowth in a premature infant, persisting postnatally	Typical craniofacial features including hypertelorism and micrognathia with thin hair	Limb/skin anomalies with mixed tone abnormalities and reflex hyperactivity	Neurological sequelae including mild intellectual disability and periventricular leukomalacia	Maternal/fetal complications with a likely pathogenic de novo EZH2 variant and gingival hypertrophy	Maternal/fetal complications with a likely pathogenic de novo EZH2 variant and gingival hypertrophy
2021	A case of Weaver syndrome caused by *EZH2* gene variation	Significant overgrowth with accelerated skeletal maturation and rapid postnatal growth	Typical craniofacial features including hypertelorism and a protruding forehead	Limb/skeletal features with finger pads, gait issues, and normal muscle function	Neurodevelopmental impairments with speech issues and poor intellectual response	Multiple surgical/medical histories with neurological and cardiac imaging abnormalities	Pathogenic de novo EZH2 p.K574E variant identified via whole-exome sequencing
2024	A case of Weaver syndrome caused by *EZH2* gene variation and literature review	Mild growth delay (possibly infection-related) consistent with potential Weaver syndrome growth traits	Typical craniofacial features including hypertelorism and large ears	Limb/skin anomalies with early fontanel closure and increased lower limb tone	Global developmental delay with feeding/swallowing issues and normal neuroimaging	Severe respiratory complications (with new diaphragmatic anomaly) and minor cardiac defect	De novo likely pathogenic EZH2 frameshift variant identified via whole-exome sequencing

AML = xxx, *EZH2 = Enhancer of Zeste Homolog 2*, HLH = xxx, ITD = xxx, NSD1 = nuclear receptor binding SET domain protein 1.

### 3.1. Clinical phenotype

Weaver syndrome is a rare congenital overgrowth disorder characterized by significant clinical heterogeneity. Its phenotype often overlaps with other overgrowth syndromes, such as Sotos syndrome, Beckwith-Wiedemann syndrome, and Marfan syndrome, making differential diagnosis challenging.^[7,8]^ Differentiating Weaver syndrome from Sotos syndrome is particularly crucial. Sotos syndrome, resulting from pathogenic variants in the *NSD1* (*Nuclear Receptor Binding SET Domain Protein 1*) gene, is also characterized by overgrowth, advanced bone age, macrocephaly, and intellectual disability. Neuroimaging can aid in the differential diagnosis; in Sotos syndrome, the most common findings are an enlarged trigone of the lateral ventricles, followed by prominent occipital horns and ventriculomegaly, which can facilitate an early diagnosis.^[9]^ The phenotypic overlap among these syndromes may stem from interactions within shared or related genetic regulatory pathways, which collectively contribute to a group of disorders primarily characterized by overgrowth. The absence of universally accepted diagnostic criteria, compounded by its rarity, poses significant challenges for the clinical diagnosis of Weaver syndrome. Consequently, diagnosis is often delayed until after the age of 2 or even into adulthood. The incidence of Weaver syndrome is approximately 3 times higher in males than in females. While the underlying mechanism for this sex bias is unclear, it has been hypothesized that females may present with a milder phenotype, leading to underdiagnosis.^[6]^ The cardinal clinical features of Weaver syndrome include prenatal and postnatal overgrowth, advanced bone age, and characteristic facial dysmorphism. These facial features typically include a broad forehead, almond-shaped palpebral fissures, a pointed chin with a prominent horizontal crease, and large, low-set ears. Other common findings are hypertonia, camptodactyly, and a range of cognitive impairments.^[1]^ Neonatal presentations can include unexplained hyperbilirubinemia, structural brain abnormalities (e.g., ventriculomegaly, cerebellar hypertrophy),^[10,11]^ and congenital heart defects.^[12]^ As more cases are documented, the known phenotypic spectrum has expanded to include features such as polydactyly,^[13]^ significant oral and dental problems (e.g., severe plaque and calculus, gingival edema, malocclusion, maxillary atresia, micrognathia, macroglossia, and bifid uvula),^[14]^ and congenital diaphragmatic eventration.^[3]^ The expression of these clinical features is highly variable, and not all patients present with the classic signs, particularly in early life. For instance, Ardinger et al described a cohort of 7 patients in 1986, some of whom displayed the characteristic facial features without overgrowth or intellectual disability; 1 patient did not develop these facial characteristics until adulthood.^[6]^ Furthermore, while overgrowth is considered a hallmark feature, it is not universally present from birth. Shimura et al reported a case in 1979 of a patient who did not exhibit overgrowth neonatally but later developed the typical craniofacial features and camptodactyly by 19 months of age.^[15]^ This clinical heterogeneity underscores the variability of the syndrome’s presentation and may be correlated with the location and type of the underlying genetic variant.

### 3.2. Pathogenesis

Pathogenic variants in the *EZH2* gene are the principal cause of Weaver syndrome. Located on chromosome 7q36.1, the *EZH2* gene spans approximately 7.7 kb and encodes the EZH2 protein.^[16]^ This protein is the core catalytic subunit of the polycomb repressive complex 2. EZH2 functions as a histone methyltransferase, specifically catalyzing the trimethylation of histone H3 at lysine 27 (H3K27me3).^[17]^ This epigenetic mark is critical for transcriptional silencing and chromatin remodeling, thereby playing a fundamental role in gene expression regulation. Consequently, pathogenic variants in EZH2 are thought to disrupt PRC2 function, leading to dysregulated cell differentiation and development. The EZH2 protein comprises several functional domains, including WD40-binding domains, SANT domains, a cysteine-rich (CXC) domain, and a C-terminal SET domain.^[18,19]^ The highly conserved SET domain mediates H3K27 methylation and is therefore an essential regulator of gene expression.^[20]^ Furthermore, through its methyltransferase activity, EZH2 regulates multiple growth-related signaling pathways, including Wnt, Notch, and TGF-β. Disruption of EZH2 function can lead to aberrant signaling. For instance, dysregulation of the Wnt signaling pathway – a key regulator of cell proliferation and tissue development – can result in the abnormal activation of Wnt target genes (e.g., *c-myc*, *cyclin D1*), promoting excessive cell proliferation and tissue overgrowth.^[21]^ In addition to *EZH2*, pathogenic variants in other core PRC2 subunits, namely *EED* and *SUZ12*, have also been implicated in Weaver syndrome, as these proteins are also integral to the complex’s methyltransferase activity.^[22]^ For example, Gibson et al reported a case caused by a variant in *EED* in 2015.^[23]^ Imagawa et al described a case linked to an *SUZ12* variant in 2017.^[24]^ Patients with variants in these genes also present with varying degrees of prenatal and postnatal overgrowth, craniofacial dysmorphism, skeletal abnormalities, and intellectual disability. These findings suggest a common pathogenic mechanism: variants in any of these 3 core components – *EZH2*, *EED*, or *SUZ12* – can impair the overall H3K27 histone methyltransferase activity of the PRC2 complex.^[25]^ This genetic heterogeneity underscores the importance of sequencing all 3 genes for accurate diagnosis. PRC2 is the sole enzyme complex in mammals responsible for H3K27 trimethylation,^[26]^ a modification crucial for normal cell growth and differentiation. Its dysregulation is directly linked to the sustained overgrowth characteristic of the syndrome.^[27]^ The majority (~90%) of pathogenic *EZH2* variants identified in patients with Weaver syndrome are missense mutations, with a smaller proportion being nonsense mutations or segmental deletions.^[10,28]^ In the present case, genetic analysis identified a c.2050C>T missense variant in the *EZH2* gene. This variant results in the substitution of arginine with cysteine at amino acid position 684 (p.Arg684Cys) and is consistent with previously reported pathogenic variants known to cause Weaver syndrome.^[2,10]^

### 3.3. Radiological findings

Magnetic resonance imaging radiographic abnormalities, most commonly advanced carpal bone age reflecting accelerated skeletal maturation, are frequent findings in Weaver syndrome. In contrast, cranial MRI abnormalities are reported less frequently. Among the documented cases with abnormal brain development, MRI findings have included ventriculomegaly (6 cases), polymicrogyria or pachygyria (2 cases), periventricular leukomalacia (2 cases), and cerebellar abnormalities (2 cases).^[10,11]^ The patient in this report presented with gyral malformations and hypomyelination in both cerebral hemispheres on cranial MRI (Fig. 3). The neurodevelopmental abnormalities observed in Weaver syndrome are hypothesized to stem from the dysregulation of PRC2 function caused by *EZH2* variants. PRC2 is crucial for neurodevelopment, where it regulates the survival and differentiation of neural progenitor cells and, consequently, myelination.^[27,29]^ For instance, the PRC2 component EED has been shown to regulate myelination in oligodendrocyte precursor cells by repressing the Wnt and BMP signaling pathways. Therefore, PRC2 dysregulation can disrupt the proliferation and differentiation of oligodendrocytes, leading to impaired myelination and abnormal brain development.^[30]^ While a direct causal link between specific *EZH2* variants and these MRI findings has not been definitively established, their presence can provide valuable clues for the clinical diagnosis of Weaver syndrome.

### 3.4. Diagnosis, treatment, and follow-up

As Weaver syndrome is an autosomal dominant disorder, offspring of an affected individual have a 50% chance of inheriting the pathogenic variant. However, due to variable expressivity, predicting the clinical severity in affected offspring is challenging. Therefore, for families with a known pathogenic variant, prenatal genetic counseling and diagnostic testing are important options for assessing reproductive risks.^[4]^ A significant advancement in diagnostics comes from the work of Choufani et al,^[31]^ who developed a specific DNA methylation (DNAm) “epi-signature” for Weaver syndrome by comparing the DNAm profiles of affected individuals with those of healthy controls. This epi-signature not only distinguishes patients from controls with high accuracy but can also differentiate between loss-of-function and gain-of-function *EZH2* missense variants. Furthermore, this approach can accurately classify variants of uncertain significance in *EED* and *SUZ12*, thereby identifying pathogenic variants in individuals with undiagnosed overgrowth/intellectual disability syndromes. Consequently, this *EZH2*-associated DNAm signature represents a powerful first-tier diagnostic tool, capable of improving diagnostic yield and efficiency for Weaver syndrome and other PRC2-related disorders.

Currently, there is no cure for Weaver syndrome; management is supportive and symptomatic. This involves a multidisciplinary approach, including surgical correction of skeletal deformities, physical and occupational therapy, speech therapy, and tailored educational support. Early diagnosis and intervention are crucial for optimizing developmental outcomes and improving long-term prognosis. Recent preclinical research has shown promise. For example, Gao et al^[32]^ developed a mouse model harboring the common p.Arg684C *EZH2* variant. These mice recapitulated key features of the syndrome, including skeletal overgrowth and enhanced osteogenic activity, which were linked to dysregulation of the BMP pathway and osteoblast differentiation. Notably, inhibiting the opposing H3K27 demethylases, KDM6A and KDM6B, reversed the osteogenic phenotype at both the transcriptional and cellular levels. This groundbreaking study suggests that targeting epigenetic regulators holds therapeutic potential for Weaver syndrome, warranting further investigation. Long-term, comprehensive follow-up is essential for managing the condition. This includes close monitoring of physical and neurodevelopmental progress, especially during infancy, with prompt referral for assessment and intervention as needed. Given that somatic *EZH2* variants are implicated in various cancers, particularly hematological malignancies,^[33]^ tumor surveillance is an important consideration. One study reported a 4.5% incidence of malignancy among 48 individuals with Weaver syndrome and *EZH2* variants.^[10]^ Although formal tumor surveillance protocols have not yet been established for Weaver syndrome, the known association between EZH2 and malignancy suggests that a heightened clinical awareness and consideration of age-appropriate cancer screening may be prudent.

During the neonatal period, this patient presented with intrauterine overgrowth and was diagnosed with macrosomia, with a birth weight of 5.04 kg and both head circumference and body length exceeding the 97th percentile. This was accompanied by characteristic facial features, including a broad forehead, hypertelorism, epicanthic folds, a flattened nasal bridge, and low-set ears. Additional findings included hyperbilirubinemia and a PDA. However, due to the rarity of the syndrome in China, its significance was overlooked by clinicians at that time. During long-term follow-up, the patient exhibited persistent overgrowth, intellectual disability, and cranial MRI findings of cerebral gyral malformations and bilateral hemispheric hypomyelination, consistent with the clinical manifestations of Weaver syndrome. Ultimately, whole-exome sequencing confirmed the diagnosis of Weaver syndrome at the age of 2 years. This mirrors the first reported case in China, where a female patient also did not display typical neonatal signs and was not diagnosed early. As she grew older, the gradual appearance of intellectual disability, distinctive facial features, prominent fetal finger pads, toe flexion contractures, and abnormal vision prompted clinical attention, leading to a genetic diagnosis at 9 years and 2 months.^[5]^ Therefore, clinicians should be vigilant for these clinical features in patients with suspected Weaver syndrome and pursue genetic testing for a definitive diagnosis when necessary. Furthermore, long-term follow-up is crucial.

The timely and accurate diagnosis of Weaver syndrome in clinical practice remains challenging, and gene sequencing is crucial for a definitive diagnosis. Cases of overgrowth, even without the typical facial features of Weaver syndrome, should be carefully assessed for this possibility.

In addition to overgrowth and distinctive facial features, our patient also presented with neonatal hyperbilirubinemia and hyperthyrotropinemia on initial admission. The specific mechanisms underlying these 2 clinical manifestations in Weaver syndrome remain incompletely understood.^[34]^

The occurrence of unexplained neonatal hyperbilirubinemia is not an isolated finding in Weaver syndrome. Tatton-Brown et al^[10]^ reported that approximately 15% of individuals with Weaver syndrome experience prolonged neonatal jaundice. This is speculated to be related to the abnormal regulation of hepatic metabolic pathways by EZH2. Mutations in the *EZH2* gene may impair the normal differentiation and function of hepatocytes, leading to disordered bilirubin metabolism and resultant jaundice. Nevertheless, the specific molecular mechanisms and pathophysiological processes require further investigation.

As a multisystem overgrowth syndrome, Weaver syndrome is often accompanied by endocrine axis abnormalities. The transient increase in thyroid-stimulating hormone in this case is presumed to result from an increased demand for thyroid hormones due to exceptionally rapid growth, coupled with a temporary insufficiency in hormone synthesis. While abnormal epigenetic regulation is known to affect thyroid function in certain genetic syndromes, more clinical and basic research is needed to support this association specifically in Weaver syndrome.

Based on our findings, we recommend that clinicians maintain a high index of suspicion for Weaver syndrome in neonates presenting with macrosomia, particularly when traditional risk factors are absent and the condition is accompanied by dysmorphic facial features and unexplained hyperbilirubinemia. Prompt targeted genetic testing is crucial for establishing a definitive diagnosis, which in turn facilitates early intervention and supportive care, ultimately improving the patient’s quality of life. Furthermore, the detailed reporting of molecular and clinical data from additional cases is essential for deepening the collective understanding of Weaver syndrome. Such contributions will expand the known genotypic and phenotypic spectrum of this rare disorder, leading to improved recognition and optimized patient management.

Weaver syndrome is a rare genetic disorder characterized by a triad of persistent overgrowth, distinctive craniofacial features, and developmental delay, particularly affecting intellectual and language faculties. Motor development may also be impaired. Associated clinical findings can include prolonged neonatal jaundice, transient hyperthyrotropinemia, and congenital heart disease. The syndrome is predominantly caused by de novo missense mutations in the *EZH2* gene, with a minority of cases attributed to mutations in *EED* or *SUZ12*. Effective management necessitates long-term multidisciplinary follow-up. While early symptomatic and therapeutic interventions can ameliorate certain functional deficits, patients carry an elevated risk of tumorigenesis that requires surveillance.

## 4. Conclusion

This paper presents a comprehensive case of Weaver syndrome caused by an *EZH2* gene mutation, confirmed through detailed clinical analysis and genetic sequencing. Our report highlights the complex and sometimes subtle clinical manifestations that can present diagnostic challenges, thereby underscoring the indispensable role of genetic analysis in the diagnosis of rare diseases. In this context, we have also summarized the current understanding of the pathogenesis, radiological features, and management strategies for Weaver syndrome. The key takeaway from our case is the critical importance of maintaining suspicion for Weaver syndrome in any instance of overgrowth – even in the absence of classic facial features – and the necessity of long-term follow-up to monitor disease progression and associated comorbidities. By contributing this case, we aim to provide new insights into the genetic mechanisms and clinical diversity of Weaver syndrome.

## Author contributions

**Conceptualization:** Luyu Ren, Liqing Jiang, Huabin Wang, Yan Li, Xueyun Ren.

**Data curation:** Luyu Ren, Liqing Jiang, Xiufang Jiang, Huabin Wang, Xueyun Ren.

**Investigation:** Luyu Ren, Liqing Jiang, Xiufang Jiang, Huabin Wang, Yan Li.

**Methodology:** Luyu Ren, Liqing Jiang, Xiufang Jiang, Huabin Wang, Yan Li.

**Writing** – **original draft:** Luyu Ren.

**Writing** – **review & editing:** Xueyun Ren.
